# Prophylaxis of Acute Attacks with a Novel Short-term Protocol in Hereditary Angioedema Patients Requiring Periodontal Treatment

**DOI:** 10.3290/j.ohpd.a42740

**Published:** 2020-07-04

**Authors:** Luca Ramaglia, Gaetano Isola, Giovanni Matarese, Maria Bova, Paolina Quattrocchi, Vincenzo Iorio-Siciliano, Agostino Guida

**Affiliations:** a Associate Professor, Department of Neurosciences, Reproductive and Odontostomatological Sciences, University of Naples ‘Federico II’, Italy. Study conception and design, analysis and interpretation of data, drafting the article.; b Junior Assistant Professor Department of General Surgery and Surgical-Medical Specialties, University of Catania, Catania, Italy. Study conception and design, analysis and interpretation of data, critical revision and interpretation of data, drafting the article.; c Associate Professor, Department of Biomedical, Odontostomatological Sciences and of Morphological and Functional Images, University of Messina, Messina, Italy. Study conception and design, drafting the article.; d Professor, Department of Translational Medical Sciences, University of Naples ‘Federico II’, Naples, Italy. Acquisition of data, drafting the article.; e Specialist, Department of Clinical and Experimental Medicine, University of Messina, Messina, Italy. Acquisition of data, drafting the article.; f Adjunct Professor, Department of Neurosciences, Reproductive and Odontostomatological Sciences, University of Naples ‘Federico II’, Naples, Italy. Analysis and interpretation of data, drafting the article.; g Resident, Department of Neurosciences, Reproductive and Odontostomatological Sciences, University of Naples ‘Federico II’, Naples, Italy. Critical revision and interpretation of data, drafting the article.

**Keywords:** chronic periodontitis, complement c1 inhibitor protein, dental scaling hereditary angioedema, periodontal debridement

## Abstract

**Purpose:**

C1-inhibitor (C1-INH) related hereditary angioedema (C1-INH-HAE) is a rare pathological condition caused by a deficiency or a functional alteration of serum protein C1-INH. Clinical manifestations are represented by recurrent, potentially life-threatening episodes of cutaneous or mucosal oedema. The present study analysed the effectiveness of a specific short-term prophylaxis protocol for the management of C1-INH-HAE patients requiring chronic periodontitis treatment.

**Materials and Methods:**

Ten consecutive C1-INH-HAE patients with mild to moderate chronic periodontitis were treated by non-surgical periodontal therapy with a full-mouth scaling approach (FMS) in two sessions 24 h apart. All patients underwent a short-term prophylaxis protocol of acute attacks based on the association of attenuated androgen (danazol), from 5 days before the first FMS session to 2 days after the second FMS session, and C1-INH concentrate given 1 h before the first FMS session. Patients were examined for periodontal changes over a 6-month period.

**Results:**

None of patients developed complications or oedema up to 1 week postoperatively. Compared to baseline, scaling and root planing (SRP) treatment yielded, at 6 months, a statistically significant improvement in probing depth (PD) (baseline: 5.24 mm ± 0.85 mm vs 6 months: 2.96 ± 0.31 mm), clinical attachment level (CAL) (baseline: 5.46 ± 0.81 vs 6 months: 3.89 ± 0.38 mm), full-mouth bleeding score (FMBS) (baseline: 27.6 ± 2.2% vs 6 months: 18.5 ± 2.1%) and in full-mouth plaque score (FMPS) (baseline: 28.6 ± 2.4% vs 6 months: 21.66 ± 3.3%).

**Conclusions:**

This study showed the clinical effectiveness of the reported prophylaxis protocol in preventing acute attacks in HAE patients requiring non-surgical periodontal treatment, with no complications up to 1 week after FMS.

Angioedema (AE) is described as localised and self-limiting oedema of subcutaneous and submucosal tissues caused by a temporary increment in vascular permeability due to the release of vasoactive mediators. Recurrent angioedema without significant wheals should be diagnosed as a distinct disease, hereditary (HAE)^24,39^ or acquired (AAE).^12^ Different types of HAE, caused by different genetic factors, have been described. The deficiency or malfunction of the serum complement protein C1-inhibitor (C1-INH) may cause a rare variant of HAE (C1-INH-HAE) with a reduced prevalence varying from 1.09/100,000 to 1.51/100,000 inhabitants.^10,42,46^ When C1-INH-HAE is caused by a deficiency of serum C1-INH, it is commonly referred to as type I C1-INH-HAE; type II C1-INH-HAE patients show regular serum C1-INH levels but there is a functional alteration of this protein.^12^ C1-INH is the main regulator of the early steps of the complement classic pathway.^14^ Indeed, in patients with C1-INH-HAE, C1 uncontrollably activates the complement system and subsequent activation of other inflammatory mediators, mainly bradykinin, occurs.^15^ The result is the onset of oedema, which can involve any cutaneous or mucosal tissue, representing the main clinical manifestation of this pathology. In type I C1-INH-HAE, which includes 85% of patients, plasmatic values of C1-INH protein are 5–30% of the normal range and are associated with low plasmatic levels of complement factor 4 (C4), an early C1-activated factor indicative of the classical pathway status. In HAE type II, plasmatic values of C1-INH are normal or elevated^5^ but the protein is not functional and the plasmatic levels of C4 are similarly low. Diagnosis of HAE type I and II is thus based on clinical symptoms, especially cutaneous oedema of face, lips, extremities, genitals or attacks of abdominal pain with nausea and vomiting, on a positive family history and on low plasmatic levels of C4 and antigenic and/or functional C1-INH. Larynx oedema may even be life-threatening because of upper airway obstruction.^6,41^

Angioedema episodes may occur spontaneously or be induced by trigger factors such as infections, drug treatments, hormonal changes, severe heat or cold, psychological stress and local trauma including dental procedures or local anesthesia.^2,6,41^

The major concern to dental practitioners is that dental treatment can provide a trigger for an episode of HAE. It has been previously reported that this treatment can be as simple as obtaining dental impressions or pulpal excavation.^47^ It can result in potentially fatal laryngeal swelling, commonly occurring 24–48 h post-treatment, with airway obstruction leading to death.^7^ There are few case reports published in the periodontal literature related to angioedema^17^; Peacock et al presented a case report in which, during periodontal surgical procedures, a patient developed angioedema of the upper lip with no impingement of the airway at any time.^35^

Treatment of C1-INH-HAE patients is thus divided into three modalities: acute attack therapy, long-term and short-term prophylaxis of acute attacks.

The resolution of acute attacks of angioedema can be obtained by modulating the bradykinin effect with plasma-derived C1-INH40 or the bradykinin receptor antagonist icatibant. In the past, concentrate or fresh-frozen plasma (FFP) was also used.^40^

Long-term prophylaxis aims to reduce the frequency and severity of the attacks and is indicated in patients who show significant and/or frequent episodes of angioedema. Drugs used for long-term prophylaxis include synthetic attenuated androgens, which increase the liver production of C1-INH protein,^9^ antifibrinolytic agents when androgens are contraindicated or ineffective,^24^ and C1-INH concentrate.

When HAE patients are at a high risk of developing acute attacks, as it may occur during dental procedures, short-term prophylaxis is indicated; this is performed mainly with C1-INH concentrate administered intravenously 1 h before the procedure. The dose of 20 UI/kg is the most common recommended prophylaxis regimen.^8^ Alternatively, short-term prophylaxis may be carried out with attenuated androgens^18,23,32^ such as danazol at a dose of 600 mg/day per os from 5 days before the procedure to the next 2 days.^25,36^ Other drugs, like antifibrinolytic agents^32,37,44^ and FFP^1,18,32^ have been used in short-term prophylaxis before oral surgery procedures. Despite pharmacological prophylaxis, some patients develop peri/postoperative oedema, as reported in several studies.^8,18^ As any dental or oral procedure may provoke acute attacks; HAE patients requiring dental treatment must be accurately evaluated and subjected to a preoperative prophylaxis to reduce the risk of subsequent attacks.

The management of HAE patients affected by periodontal disease may be a major issue as, to date, there are no definite guidelines or reports. Any type of periodontal treatment may represent a trigger factor for acute HAE attacks. Anti-infective non-surgical periodontal therapy, in conjunction with oral hygiene motivation and instructions, is the primary treatment of chronic periodontitis. Three equally effective approaches of non-surgical periodontal therapy are reported in the literature^21^: (a) conventional scaling and root planing (SRP), performed at one quadrant or sextant per week; (b) full-mouth scaling (FMS), ie, scaling and root planing of the whole mouth performed within 24 h; and (c) full-mouth disinfection (FMD), ie, scaling and root planing of the whole mouth performed within 24 h plus tongue brushing and adjunctive antiseptic administration including rinsing of the mouth, pocket irrigation and spraying of the tonsils.

In the present study, consecutive cases of HAE patients affected by chronic periodontitis and treated with FMS are reported. In order to reduce the risk of acute attacks, patients were subjected to a novel protocol of short-term prophylaxis based on the association of attenuated androgen and C1-INH concentrate.

The purpose of the present study was to analyse the effectiveness of a specific short-term prophylaxis protocol for the management of C1-INH-HAE patients requiring chronic periodontitis treatment.

## Materials and Methods

The study was designed as a case series. From January 2014 to December 2016, 10 consecutive patients, affected by HAE and with mild to moderate chronic periodontitis, were referred to the Department of Odontostomatology of the University of Messina for appropriate treatment. The local ethical committee approved the study protocol (#919-10).

The inclusion criteria were (1) diagnosis of HAE; (2) a minimum of two teeth in each quadrant with a probing depth (PD) ≥ 5 mm; (3) ≥ 40% sites with bleeding on probing; and (4) no involvement of furcations. The exclusion criteria were: (1) periodontal therapy during the last 12 months; (2) pregnancy; (3) previous or current radiation or immunosuppressive therapy; (4) medication by anti-inflammatory and immunosuppressive drugs; (5) previous history of excessive drinking; (6) smoking; and (7) class II and III tooth mobility.

The patients were carefully evaluated through a clinical examination and laboratory tests, including C1-INH and C4 plasmatic levels, and C1-INH activity. Furthermore, periodontal clinical data were recorded in all patients and included PD, clinical attachment level (CAL), gingival recession (GR), full-mouth plaque score (FMPS)^34^ and full-mouth bleeding score (FMBS),^13^ that were registered at six sites per tooth (ie, distobuccal, buccal, mesiobuccal, mesiolingual, lingual, distolingual) using a manual periodontal probe (PCP-UNC 15, Hu-Friedy, Chicago, IL, USA). CAL was defined as the distance from the cementoenamel junction (CEJ) to the bottom of the sulcus or periodontal pocket and was calculated as the sum of pocket probing depth and GR measurements. Pocket probing depth was defined as the distance from the free gingival margin to the bottom of the sulcus or periodontal pocket. Gingival recession was defined as the distance from the CEJ to the free gingival margin.

In the absence of guidelines, and taking into account the systemic condition, patients were prepared for anti-infective non-surgical periodontal therapy with an FMS protocol associated with HAE short-term prophylaxis in order to reduce the risk of acute attacks during and after the periodontal procedures. Detailed information about the treatment protocols were given and the patients signed informed consent forms.

To ensure patients’ compliance and tolerance, FMS was scheduled in two separate sessions under local anaesthesia within 24 h. Prior to FMS, proper oral hygiene motivation and instructions were given to all patients.

After immunological consultation, all patients received a specific short-term HAE protocol based on attenuated androgen such as danazol 600 mg/day (Danatrol 200 mg t.i.d, Sanofi, Italy) starting from 5 days before the first FMS session to 2 days after the second FMS session, in conjunction with C1-INH concentrate (Berinert P, CSL Behring, Lev Pharmaceuticals, NY, USA). The latter was given by slow intravenous perfusion (4 ml/min) at a recommended dose of 20 UI/kg, 1 h before the first FMS procedure. The patients who were under long-term prophylaxis adjusted their usual therapy in order to follow the specific short-term prophylaxis protocol.

Each FMS session was performed by an experienced periodontist under local anaesthesia with 2% mepivacaine with 1:100,000 epinephrine.

The two FMS sessions were performed on two subsequent days (day 1 and day 2) on a one-inpatient basis, with an active fast-track to the emergency room in case of a severe attack of angioedema such as pharyngeal/laryngeal oedema and airway obstruction. Patients were recommended to stay at the hospital for at least 6 hours after the end of the FMS session. 0.12% chlorhexidine gluconate mouthrinse was prescribed twice-daily and short-term clinical controls were scheduled for the day after the second FMS session (day 3), and 6 days after the first session (day 7).

The second FMS sessions were performed in all patients without a second perfusion of C1-INH concentrate, as this medication has a half-life of more than 24 hours.^11,12,30^

## Results

Clinical features of the patients are presented in Table 1. The sample of the study was composed by four males and six females, aged 40 to 60 years (mean age 49 years). Seven patients were affected by type I C1-INH-HAE and three patients suffered from type II C1-INH-HAE. In the whole sample, the mean duration of disease onset was 27.2 ± 7.9 years. Three patients, due to the frequency and severity of HAE attacks, were being given regular long-term prophylaxis, based on attenuated androgens.

**Table 1:** Patients’ clinical features

Patient	Sex	Age (Years)	HAE Type	Comorbidities	Long-Term Prophylaxis	HAE Symptoms
1	F	40	I	/	/	Oedema of the lips, abdominal attacks
2	F	55	II	Depression	Attenuated androgens	Oedema of the extremities, abdominal attacks
3	M	60	I	Hypertension, diabetes mellitus	/	Oedema of the extremities and of the upper respiratory tract
4	M	47	I	/	/	Abdominal attack, oedema of the face
5	F	53	II	/	Attenuated androgens	Abdominal attack, oedema of the extremities
6	F	42	I	Diabetes mellitus	/	Abdominal attack, oedema of the face
7	M	51	I	/	/	Oedema of the lips, abdominal attacks
8	F	48	I	Hypertension		Abdominal attack, oedema of the extremities
9	M	44	I	/	Attenuated androgens	Oedema of the lips, abdominal attacks
10	F	50	II	Hypertension		Abdominal attack, oedema of the extremities

A summary of periodontal clinical parameters is reported in Table 2. Means with standard deviations (± SD) were determined to describe the data. SRP yielded, at 6 months of follow-up, a statistically significant improvement in PD (baseline: 5.24 mm ± 0.85 mm vs 6 months: 2.96 ± 0.31 mm), CAL (baseline: 5.46 ± 0.81 vs 6 months: 3.89 ± 0.38 mm), GR (baseline: 0.12 ± 0.21 mm vs 6 months: 0.41 ± 0.23 mm), FMBS (baseline: 27.6 ± 2.2% vs 6 months: 18.5 ± 2.1%), as well as the FMPS (baseline: 28.6 ± 2.4% vs 6 months: 21.66 ± 3.3%) (Table 2).

**Table 2:** Patients’ periodontal clinical parameters

	PD (mm)	CAL (mm)	GR (mm)	FMPS (%)	FMBS (%)
Baseline	5.24 ± 0.85	5.46 ± 0.81	0.12 ± 0.21	28.6 ± 2.4	27.6 ± 2.2
3 months	3.52 ± 0.91	4.12 ± 0.79	0.23 ± 0.18	25.4 ± 2.1	20.9 ± 1.9
6 months	2.96 ± 0.31	3.89 ± 0.38	0.41 ± 0.23	21.66 ± 3.3	18.5 ± 2.1

A summary of patients’ laboratory data and postoperative complications after the FMS sessions is presented in Table 3. On day 2, C1-INH and C4 plasmatic levels were into physiological ranges and higher than their values at diagnosis as an effect of the pharmacological prophylaxis therapy.

**Table 3:** Summary of patients’ laboratory and clinical data

Patient	C1-INH at diagnosis (g/L)	C1-INH at day 2 (g/L)	C4 at diagnosis (g/L)	C4 at day 2 (g/L)	C1-INH activity at diagnosis	Postoperative complications
1	0.17	0.24	0.06	0.12	Reduced	None
2	0.57	0.58	0.06	0.11	Reduced	None
3	0.19	0.25	0.08	0.12	Reduced	None
4	0.15	0.21	0.05	0.10	Reduced	None
5	0.45	0.49	0.06	0.10	Reduced	None
6	0.17	0.28	0.06	0.11	Reduced	None
7	0.19	0.24	0.05	0.11	Reduced	None
8	0.18	0.26	0.05	0.10	Reduced	None
9	0.50	0.52	0.08	0.12	Reduced	None
10	0.16	0.22	0.06	0.12	Reduced	None

(normal range: C1-INH 0.21–0.39 g/L; C4 0.10–0.40 g/L; C1-INH activity: > 70%)

**Fig 1:**
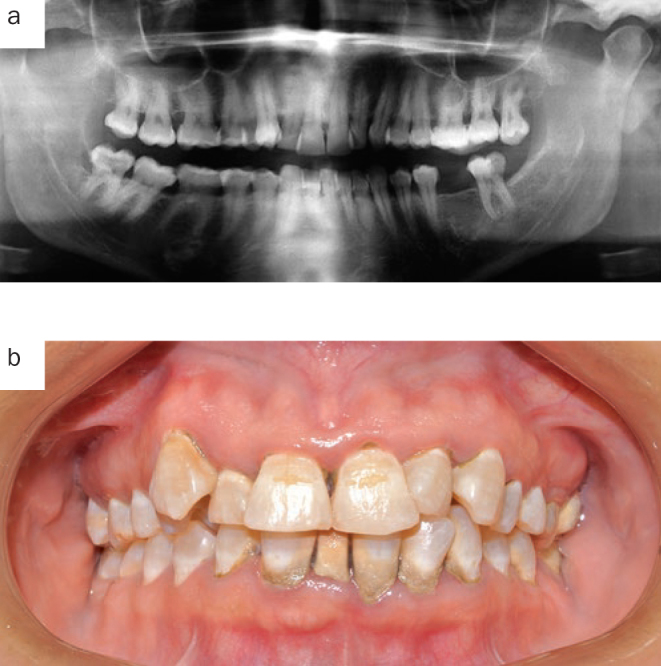
Patient 1, panoramic dental radiograph (a) and initial intraoral condition (b).

**Fig 2:**
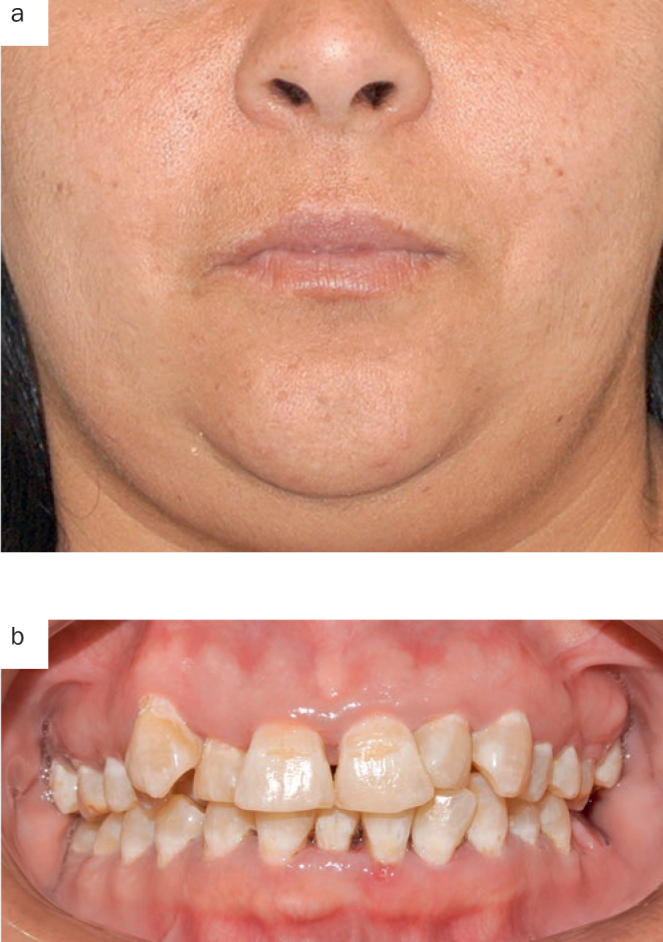
Patient 1, extraoral (a) and intraoral (b) condition at day 3; no sign of facial swelling/oedema.

**Fig 3:**
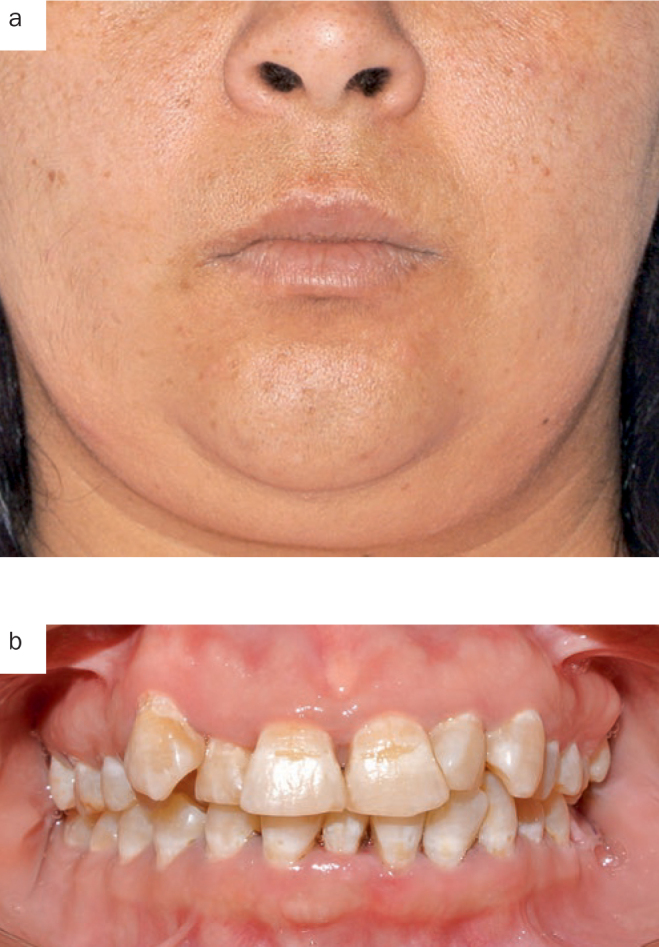
Patient 1, extraoral (a) and intraoral (b) condition at day 7; no sign of facial swelling/oedema.

No acute HAE attack was observed in any patient during the FMS sessions or the following 6-hour hospital stay, nor subsequently. After completion of the FMS procedure, none of the 10 patients showed any sign of facial swelling/oedema or any other HAE symptom up to 6 days after the first session (day 7), as reported by the patients and as observed at the clinical evaluations scheduled on day 3 and day 7 (Figs 1, 2 and 3).

## Discussion

C1-INH-HAE is a rare autosomal pathological condition caused by deficiency or inactivity of serum C1-INH protein. This alteration may lead to an inappropriate or excessive activation of the complement classical pathway and to an excess of bradykinin release.^15,16^ The subsequent increase in vascular permeability induces local oedema, which represents the main clinical manifestation of this syndrome. Episodes may occur spontaneously, or be induced by trigger factors including dental procedures or local anesthesia.^2,6,28,41^

Short-term prophylaxis of acute HAE attacks is indicated in the case of dental procedures or oral surgery since these may induce swelling of the lips, the face, the tongue or even a laryngeal oedema with upper airway obstruction. Cases of death by asphyxiation due to laryngeal oedema following oral procedures in HAE patients have been described.^6,41^

In the present study, we report 10 consecutive cases of C1-INH-HAE patients affected by mild to moderate chronic periodontitis, and thus requiring periodontal therapy.^20,31,43^ The patient sample is sufficiently representative, considering the estimated prevalence of HAE in the global population^48^; this is of particular interest, since seven patients were affected by type I C1-INH-HAE and three patients by type II C1-INH-HAE.

Any type of periodontal treatment may represent a trigger factor for acute HAE attacks. Anti-infective non-surgical periodontal therapy, in conjunction with oral hygiene motivation and instructions, is the primary treatment of chronic periodontitis.^22,26,27,29^ In the absence of guidelines, and taking into account the patients’ systemic condition, FMS was considered the most suitable anti-infective non-surgical periodontal therapy and preferred to SRP and FMD.

The mechanisms underlying the role of periodontal tissue damage in patients with HAE involving downregulation of immune responses^45^ or upregulation by production of glucocorticoids.^4^ During stressful periods, the hypothalamic–pituitary–adrenal axis increases its release of corticotrophin-releasing hormone (CRH) and this stimulates the pituitary gland to release adrenocorticotrophic hormone (ACTH) and β-endorphin. Moreover, in response to stress during HAE, there is the releasing of interleukin-1 (IL-1) able to stimulate CRH release from the hypothalamus as well as from lymphocytes.^3^ β-endorphin has been shown to increase dramatically during acute episodes of HAE by the pituitary.^37-39^ The excessive β-endorphin release centrally during stress linked to HAE, and locally from IL-1 stimulation of periodontal inflammatory cells, may disrupt the balance between proinflammatory (complement-mediated) and anti-inflammatory events, determining the periodontal tissue destruction. The subsequent release of reactive oxygen species (ROS), proteolytic enzymes and other inflammatory mediators (eg, PGE2) by accumulated phagocytes (and fibroblasts) in areas of high antigenic load may exceed a threshold consistent with tissue stability.^19^

In the present study, compared to baseline, SRP treatment yielded in all enrolled patients a significant improvement in all periodontal parameters evaluated such as PD, CAL, GR, FMBS and FMPS at 6 months, without any side effects. SRP was discarded as it would have required several short-term prophylaxis drug treatments repeated over a period of weeks. As for FMS and FMD approaches, they have shown a similar clinical efficacy compared to SRP in a recent systematic review^21^ in which the authors concluded that the decision to select one approach over another may be based on patient preference and the convenience of the treatment schedule. Given the necessity of short-term prophylaxis of acute HAE attacks, FMS seemed the more suitable as it is performed in a short period of time but, compared to FMD, it avoids repeated and consistent antimicrobial administrations which, especially in the case of tonsil spraying, may play an adjunctive role as trigger factors for acute HAE attacks. As suggested, FMS was performed in two sessions within 24 hours and under local anaesthesia to ensure patients’ compliance.

Patients were evaluated before FMS procedure through an accurate clinical examination and laboratory tests. Subsequently, patients were subjected to a specific preoperative short-term HAE prophylaxis protocol based, for the first time to our knowledge, on patients requiring periodontal treatment on the association of attenuated androgens from 5 days before the procedure to the next 2 days and C1-INH concentrate administered intravenously 1 h before the first session of FMS.

Each FMS session was performed under local anaesthesia with 2% mepivacaine with 1:100,000 epinephrine. A previous report suggested that, in patients with HAE, the dental treatments should be performed under general anaesthesia in order to have a controlled setting where the life-threatening angioedema could be treated if present.^33^ However, several cases of laryngeal oedema consequent to intubation has already been demonstrated following general anesthesia.^7,38^ For this reason, in the absence of specific guidelines, and taking into account the systemic condition, patients were planned for anti-infective non-surgical periodontal therapy with an FMS approach under local anaesthesia using mepivacaine.

In all patients FMS was completed without complications and with no occurrence of acute HAE attacks within the week following the procedures.

This study showed the clinical effectiveness of the reported prophylaxis protocol in preventing acute attacks in HAE patients requiring non-surgical periodontal treatment, with no complications up to 1 week after FMS. The results of the present study are promising but demand further research with a larger sample to better understand the efficacy of the study protocol and to gather more data on specific periodontal procedures likely to trigger HAE attacks. Moreover, further studies should be performed also to understand if this protocol based on an association of attenuated androgens and C1-INH concentrate may be more effective than protocols based on one of the two medications alone.

## Conclusion

Although preoperative prophylaxis significantly reduces the risk of acute attacks, this risk cannot be completely avoided.^8^ For this reason, it is important for both the patient and the periodontist to be aware of the clinical characteristics of the acute attacks in order to identify the early symptoms and perform timely interventions in case of emergency.

The possibility of performing dental treatments free of risk of acute attacks or complications on consecutive days with the reported short-term prophylaxis protocol, based on the association of attenuated androgens and C1-INH concentrate, may be of relevant clinical interest for the treatment of C1-INH-HAE patients affected by chronic periodontitis and thus requiring periodontal treatment.

In conclusion, patients with a history of C1-INH-HAE should be subjected to an accurate clinical and laboratory examination before any periodontal procedure.
